# Efficacy and safety analysis of TACE + Donafenib + Toripalimab versus TACE + Sorafenib in the treatment of unresectable hepatocellular carcinoma: a retrospective study

**DOI:** 10.1186/s12885-023-11535-5

**Published:** 2023-10-25

**Authors:** Haohao Lu, Bin Liang, Xiangwen Xia, Chuansheng Zheng

**Affiliations:** 1 Department of Radiology, Union Hospital, Tongji Medical College, Huazhong University of Science and Technology, Jiefang Avenue #1277, Wuhan, 430022 China; 2 Hubei Province Key Laboratory of Molecular Imaging, Wuhan, 430022 China

**Keywords:** Hepatocellular carcinoma, Transarterial chemoembolization, Targeted therapy, Immunotherapy, Immune checkpoint inhibitors, Tyrosine kinase inhibitors, Interventional treatment.

## Abstract

**Objective:**

To compare the efficacy and safety of TACE combined with Donafenib and Toripalimab versus TACE combined with Sorafenib in the treatment of unresectable hepatocellular carcinoma (HCC), aiming to guide personalized treatment strategies for HCC and improve patient prognosis.

**Materials and methods:**

A retrospective analysis was conducted on the clinical data of 169 patients with unresectable advanced-stage HCC who underwent treatment at the Interventional Department of Wuhan Union Hospital from January 2020 to December 2022. Based on the patients’ treatment strategies, they were divided into two groups: TACE + Donafenib + Toripalimab group (*N* = 81) and TACE + Sorafenib group (*N* = 88). The primary endpoints were objective response rate (ORR), disease control rate (DCR), overall survival (OS), and progression-free survival (PFS) of the two groups’ tumors. The secondary endpoint was the occurrence of treatment-related adverse events in the two groups of patients.

**Results:**

The TACE + Donafenib + Toripalimab group showed higher ORR and DCR compared to the TACE + Sorafenib group (66.7% vs. 38.6%, 82.6% vs. 68.2%, *P* < 0.05). The TACE + Donafenib + Toripalimab group also demonstrated longer median progression-free survival (mPFS) (10.9 months vs. 7.0 months, *P* < 0.001) and median overall survival (mOS) (19.6 months vs. 10.9 months, *P* < 0.001) compared to the TACE + Sorafenib group. When comparing the two groups, the TACE + Sorafenib group had a higher incidence of grade 3–4 hypertension (14.8% vs. 4.9%, *P* = 0.041), higher incidence of diarrhea (all grades) (18.2% vs. 7.4%, *P* = 0.042), and higher incidence of hand-foot syndrome (all grades) (26.1% vs. 12.3%, *P* = 0.032).

**Conclusion:**

TACE combined with Donafenib and Toripalimab demonstrates superior efficacy and safety in treating unresectable HCC patients. This combination therapy may serve as a feasible option to improve the prognosis of unresectable HCC patients.

## Introduction

Hepatocellular Carcinoma (HCC) is one of the most prevalent malignant tumors worldwide and represents the primary type of liver malignancy [1]. According to the World Health Organization (WHO), as of 2020, HCC ranked sixth in terms of incidence and third in terms of mortality among all cancers globally [2]. In certain high-risk regions such as China and Southeast Asia, the incidence and mortality rates of HCC are even higher [3, 4]. The major risk factors for HCC include viral infections, dietary habits, alcohol consumption, obesity, medication, and exposure to toxins [5–7]. Due to its asymptomatic early stage and rapid progression, most patients are diagnosed at an advanced stage when surgical resection is not feasible [8, 9]. Despite the challenges associated with HCC treatment, there has been some progress in recent years. Transarterial chemoembolization (TACE) is one of the commonly used approaches for the treatment of unresectable HCC [10]. Particularly for patients classified as BCLC B stage, TACE significantly improves survival outcomes [11]. Some BCLC C stage patients also benefit from TACE in terms of survival [12, 13]. TACE delivers chemotherapy drugs directly to the tumor region via the hepatic artery and combines them with embolic agents to inhibit tumor growth [14]. However, the efficacy of single TACE treatment is limited in terms of long-term survival and disease control. Consequently, researchers have begun exploring the possibility of combining TACE with other treatment modalities to improve patient prognosis [15, 16]. In this regard, molecular targeted therapy and immunotherapy have attracted widespread attention as novel treatment strategies for HCC [17, 18]. Commonly used molecular targeted drugs include sorafenib, lenvatinib, regorafenib, apatinib, and donafenib [19]. Donafenib, a multitarget tyrosine kinase inhibitor, exerts antitumor effects by inhibiting tumor cell proliferation and angiogenesis [20]. Early clinical trials have shown that donafenib significantly prolongs progression-free survival and overall survival in patients with unresectable HCC [20]. Toripalimab, an immune checkpoint inhibitor, activates the patient’s immune system by blocking the PD-1 and PD-L1 signaling pathways, enhancing the ability to attack tumors [21]. Although clinical research on tislelizumab in HCC is still in the early stages, some studies have shown its potential in tumor control and improvement of patient prognosis [22]. For unresectable HCC patients, the combination of TACE with donafenib and toripalimab has attracted researchers’ interest. The theoretical basis for this comprehensive treatment strategy is to directly target the tumor with TACE while utilizing the molecular targeting and immune-modulating effects of donafenib and toripalimab to enhance treatment efficacy. In comparison, TACE combination with sorafenib is another commonly used treatment approach [23]. Sorafenib, another multitarget tyrosine kinase inhibitor, exerts its effects through mechanisms such as inhibiting tumor cell proliferation, anti-angiogenesis, and immune modulation [24]. Sorafenib has been widely used in the treatment of unresectable HCC patients and has shown certain efficacy. However, there is limited research data directly comparing the effectiveness of TACE combination with donafenib and toripalimab versus TACE combination with sorafenib in treating unresectable HCC. Therefore, this study aims to evaluate the efficacy and safety of these two treatment approaches by retrospectively analyzing clinical data from two patient groups. We expect that the results of this study will provide further evidence for the treatment selection of unresectable HCC patients. By comparing the clinical outcomes and safety profiles of TACE combination with donafenib and toripalimab versus TACE combination with sorafenib, we will gain a better understanding of the advantages and limitations of these treatment approaches in improving patient survival, controlling tumor progression, and minimizing adverse events. This is of significant importance in guiding personalized treatment strategies for HCC and promoting patient prognosis.

## Materials and methods

### General information

Clinical data of 169 patients with unresectable advanced-stage hepatocellular carcinoma (HCC) who received treatment at the Department of Interventional Radiology, Tongji Medical College, Huazhong University of Science and Technology, Union Hospital, from January 2020 to December 2022 were collected. Inclusion criteria were as follows: (1) Age between 18 and 70 years; (2) Diagnosis of HCC confirmed by pathological and radiological examinations [25]; (3) No previous treatment for liver cancer, including radiation therapy, chemotherapy, targeted therapy, or immunotherapy; (4) Child-Pugh liver function classification of A-B, Eastern Cooperative Oncology Group (ECOG) performance status of 0–2; (5) White blood cell count ≥ 3.0 G/L, platelet count ≥ 50 G/L, international normalized ratio (INR) ≤ 1.5; (6) Complete clinical follow-up data. Exclusion criteria were as follows: (1) Presence of primary or metastatic cancer in other sites; (2) Severe abnormalities in cardiac, pulmonary, renal, hematological, neurological, or coagulation functions; (3) Tumor volume > 70% of liver volume; (4) Allergy to iodinated contrast agents, donafenib, sorafenib, or toripalimab. Based on the treatment strategy, the patients were divided into two groups: TACE + Donafenib + Toripalimab group (*N* = 81) and TACE + Sorafenib group (*N* = 88). Baseline data were collected, including gender, age, etiology of liver cirrhosis, preoperative Child-Pugh liver function classification, ECOG performance status, BCLC tumor staging, pre-treatment total bilirubin, ALT, AST, white blood cell count, red blood cell count, and platelet count.

### Methods

#### TACE Procedure [26]

The patient was positioned supine, and the groin area was sterilized with iodine solution. Local anesthesia was administered at the puncture site using 2% lidocaine, and a 5 F vascular sheath was inserted using the Seldinger technique. A 5 F Yashiro catheter was advanced into the celiac trunk and superior mesenteric artery (or other collateral arteries if necessary) for angiography to identify the tumor-feeding arteries. Then, a 2.7 F microcatheter was selectively inserted into the tumor-feeding artery of the HCC, and a mixture of iodized oil and epirubicin was injected to form an emulsion. Finally, 300–500 μm gelatin sponge particles were injected for embolization, and the embolization endpoint was defined as the stasis of forward blood flow in the tumor-feeding artery. If an arterioportal or arteriovenous shunt was found during intraoperative angiography, the microcatheter was selectively advanced to the shunt site, and polyvinyl alcohol (PVA) particles were used to embolize and occlude the shunt before proceeding with subsequent chemoembolization. After the treatment, the catheter was removed, and the puncture site was compressed and bandaged.

Materials and drugs used for TACE included: 5 F vascular sheath (TERUMO5F-10CM, Terumo, Japan), 0.035 inch guidewire (RFGA35153M, Terumo, Japan), 5 F Yashiro catheter (Terumo, Japan), 2.7 F microcatheter (Terumo, Japan), epirubicin (GYZZ H19990280, Zhejiang Hisun Pharmaceutical Co., Ltd.), iodized oil (GYZZ H20163348, Jiangsu Hengrui Medicine Co., Ltd.).

#### Usage of Donafenib and Toripalimab

Donafenib: 200 mg, orally, twice daily.

Toripalimab: 240 mg, intravenous infusion, every 3 weeks.

#### Usage of Sorafenib

Dosage: 400 mg, orally, twice daily.

Patients underwent contrast-enhanced CT or MRI follow-up every 4–6 weeks, and the decision for further TACE treatment was based on the follow-up results.

### Observation indicators

Primary endpoints:

1. Evaluation of tumor response after treatment in both groups using the mRECIST criteria [27], including complete response (CR), partial response (PR), stable disease (SD), and progressive disease (PD).
2. Objective response rate (ORR) and disease control rate (DCR) of the tumors in both groups.
3. Overall survival (OS) and progression-free survival (PFS) in both groups.

Secondary endpoints:

1. Changes in liver function and blood routine before treatment and three months after treatment in both groups.
2. Incidence of treatment-related adverse events in both groups.

### Statistical methods

Statistical analysis was performed using SPSS 24.0 software. Categorical data were presented as frequencies (percentages), and intergroup differences were assessed using the chi-square test, including Pearson Chi-Square and Fisher’s Exact Test. Continuous data were presented as mean ± standard deviation, and intergroup differences were analyzed using the t-test. OS and PFS were displayed using Kaplan-Meier curves, and the comparison of OS and PFS between the two groups was performed using the Log-Rank test. A *p*-value of < 0.05 was considered statistically significant.

## Results

### Comparison of baseline characteristics between the two groups (see Table 1)

**Table 1:** Comparison of Baseline Characteristics between the Two Groups

			Group				
			TACE+Donafenib+Toripalimab group (*N*=81)	TACE+Sorafenib group (*N*=88)	Chi-Square Tests (*p*-value)		t-test (*p*-value)
					Fisher's Exact Test	Pearson Chi-Square	
sGender	Female	Count(%)	16(19.8%)	21(23.9%)	0.579		
	Male	Count(%)	65(80.2%)	67(76.1%)			
Etiology of cirrhosis	Hepatitis B	Count(%)	54(66.7%)	51(58.0%)		0.663	
	Hepatitis C	Count(%)	13(16.0%)	16(18.2%)			
	Alcohol	Count(%)	8(9.9%)	13(14.8%)			
	others	Count(%)	6(7.4%)	8(9.0%)			
Pre-treatment ECOG	0	Count(%)	31(38.3%)	33(37.5%)		0.869	
	1	Count(%)	35(43.2%)	41(46.6%)			
	2	Count(%)	15(18.5%)	14(15.9%)			
Pre-treatment liver function	Child A	Count(%)	46(56.8%)	48(54.5%)	0.877		
	Child B	Count(%)	35(43.2%)	40(45.5%)			
BCLC staging	B	Count(%)	22(27.2%)	28(31.8%)	0.613		
	C	Count(%)	59(72.8%)	60(68.2%)			
Age(Years)	Mean±SD		51.9±12.4	53.9±12.1			0.295
Pre-treatment bilirubin(μmol/L)	Mean±SD		18.87±7.93	17.12±7.23			0.137
Pretreatment ALT(U/L)	Mean±SD		38.6±25.5	42.8±24.8			0.276
Pretreatment AST(U/L)	Mean±SD		48.1±31.7	40.8±21.6			0.085
Pretreatment WBC(G/L)	Mean±SD		3.84±1.12	4.07±1.06			0.171
Pretreatment RBC(T/L)	Mean±SD		4.32±1.73	4.59±1.69			0.300
Pretreatment PLT(G/L)	Mean±SD		117.3±51.4	126.4±61.9			0.297

There were no significant statistical differences (*P* > 0.05) in terms of gender, age, etiology of liver cirrhosis, preoperative liver function based on Child-Pugh classification, ECOG score, BCLC stage of the tumor, pre-treatment total bilirubin, ALT, AST, white blood cell count, red blood cell count, and platelet count between the two groups.

### Comparison of blood parameters at three months after treatment in the two groups (see Table 2).

**Table 2:** Comparison of Hematological Parameters between the Two Groups after Three Months of Treatment

		Group		t-test (*p*-value)
		TACE + Donafenib + Toripalimab group (*N* = 81)	TACE + Sorafenib group (*N* = 88)	
Post-treatment bilirubin(µmol/L)	Mean ± SD	18.44 ± 7.60	19.73 ± 8.51	0.304
Post-treatment ALT(U/L)	Mean ± SD	72.6 ± 41.6	79.1 ± 30.3	0.253
Post-treatment AST(U/L)	Mean ± SD	75.8 ± 43.8	80.3 ± 32.3	0.443
Post-Treatment WBC(G/L)	Mean ± SD	5.46 ± 1.51	5.29 ± 1.56	0.483
Post-Treatment RBC(T/L)	Mean ± SD	3.73 ± 1.07	3.61 ± 1.15	0.449
Post-Treatment PLT(G/L)	Mean ± SD	79.7 ± 20.8	74.5 ± 26.7	0.157

There were no significant differences (*P* > 0.05) in terms of total bilirubin, ALT, AST, white blood cell count, red blood cell count, and platelet count at three months after treatment between the two groups.

### Evaluation of tumor response after treatment in the two groups (see Table 3)

**Table 3:** Evaluation of Tumor Response in the Two Patient Groups

			Group		Chi-Square Tests(*p*-value)	
			TACE + Donafenib + Toripalimab group(*N* = 81)	TACE + Sorafenib group(*N* = 88)	Pearson Chi-Square	Fisher’s Exact Test
Tumor response	CR	Count(%)	13(16.0%)	5(5.7%)	0.002	
	PR	Count(%)	41(50.6%)	29(33.0%)		
	SD	Count(%)	13(16.0%)	26(29.5%)		
	PD	Count(%)	14(17.4%)	28(31.8%)		
ORR		Count(%)	54(66.7%)	34(38.6%)		< 0.001
DCR		Count(%)	67(82.6%)	60(68.2%)		0.033

The proportion of patients achieving complete response (CR) and partial response (PR) after treatment was higher in the TACE + Donafenib + Toripalimab group compared to the TACE + Sorafenib group, and the proportion of patients with progressive disease (PD) was lower in the TACE + Donafenib + Toripalimab group compared to the TACE + Sorafenib group (*P* = 0.002). The objective response rate (ORR) and disease control rate (DCR) after treatment were both higher in the TACE + Donafenib + Toripalimab group compared to the TACE + Sorafenib group (P < 0.05).

### Comparison of overall survival (OS) and progression-free survival (PFS) in the two groups (see Table 4).

**Table 4:** Comparison of Overall Survival (OS) and Progression-Free Survival (PFS) between the Two Patient Groups

	Gruop	Median(months)	95% Confidence Interval		Log Rank (Mantel-Cox) (*p*-value)
			Lower Bound	Upper Bound	
PFS	TACE + Donafenib + Toripalimab group	10.9	8.7	11.3	< 0.001
	TACE + Sorafenib group	7.0	6.2	7.8	
OS	TACE + Donafenib + Toripalimab group	19.6	17.9	21.2	< 0.001
	TACE + Sorafenib group	10.9	9.8	12.0	

**Fig. 1:**
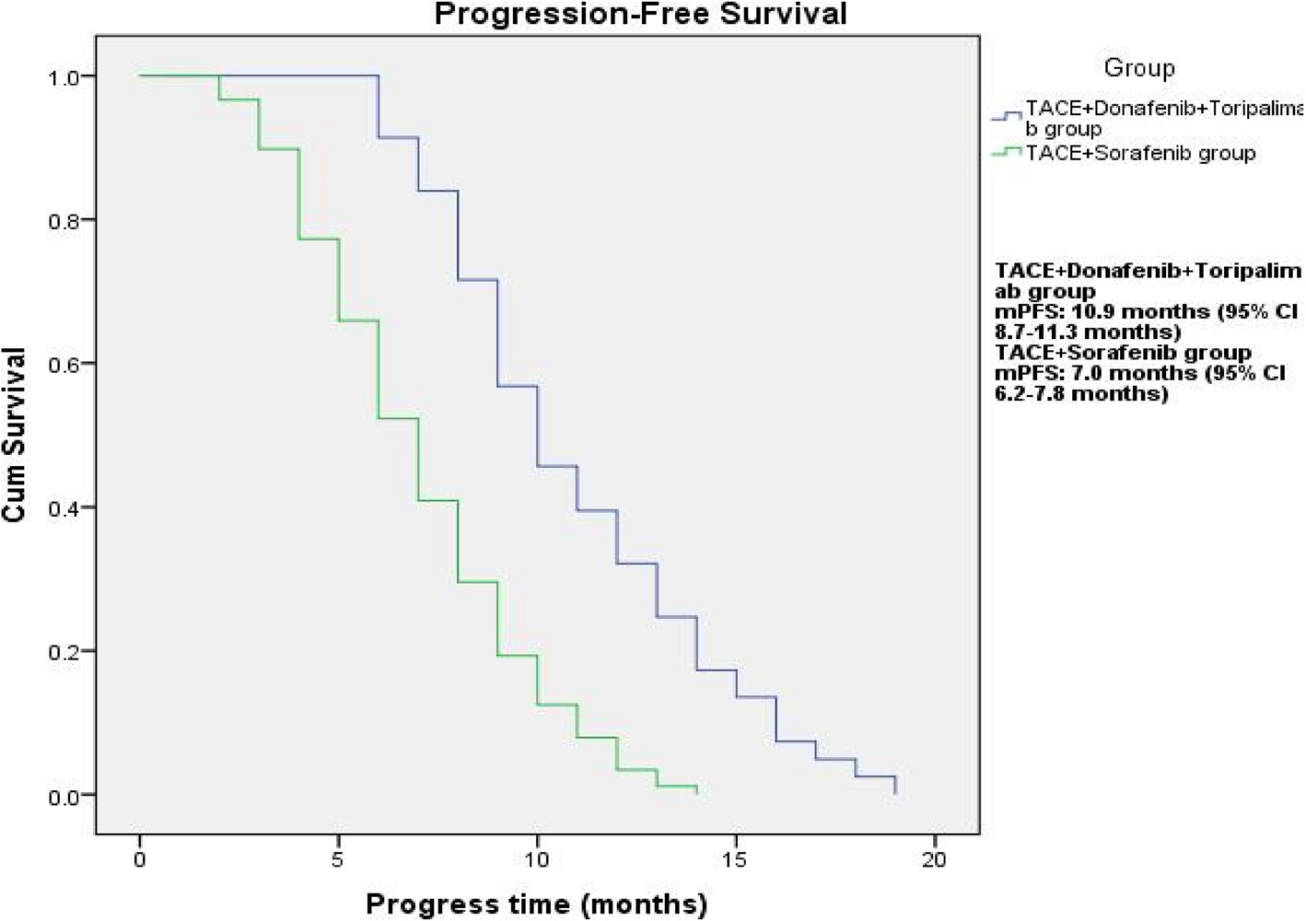
Progression-free survival time in the two groups. mPFS: TACE+Donafenib+Toripalimab group, 10.9 months (95% CI: 8.7-11.3 months); TACE+Sorafenib group, 7.0 months (95% CI 6.2-7.8 months)

**Fig. 2:**
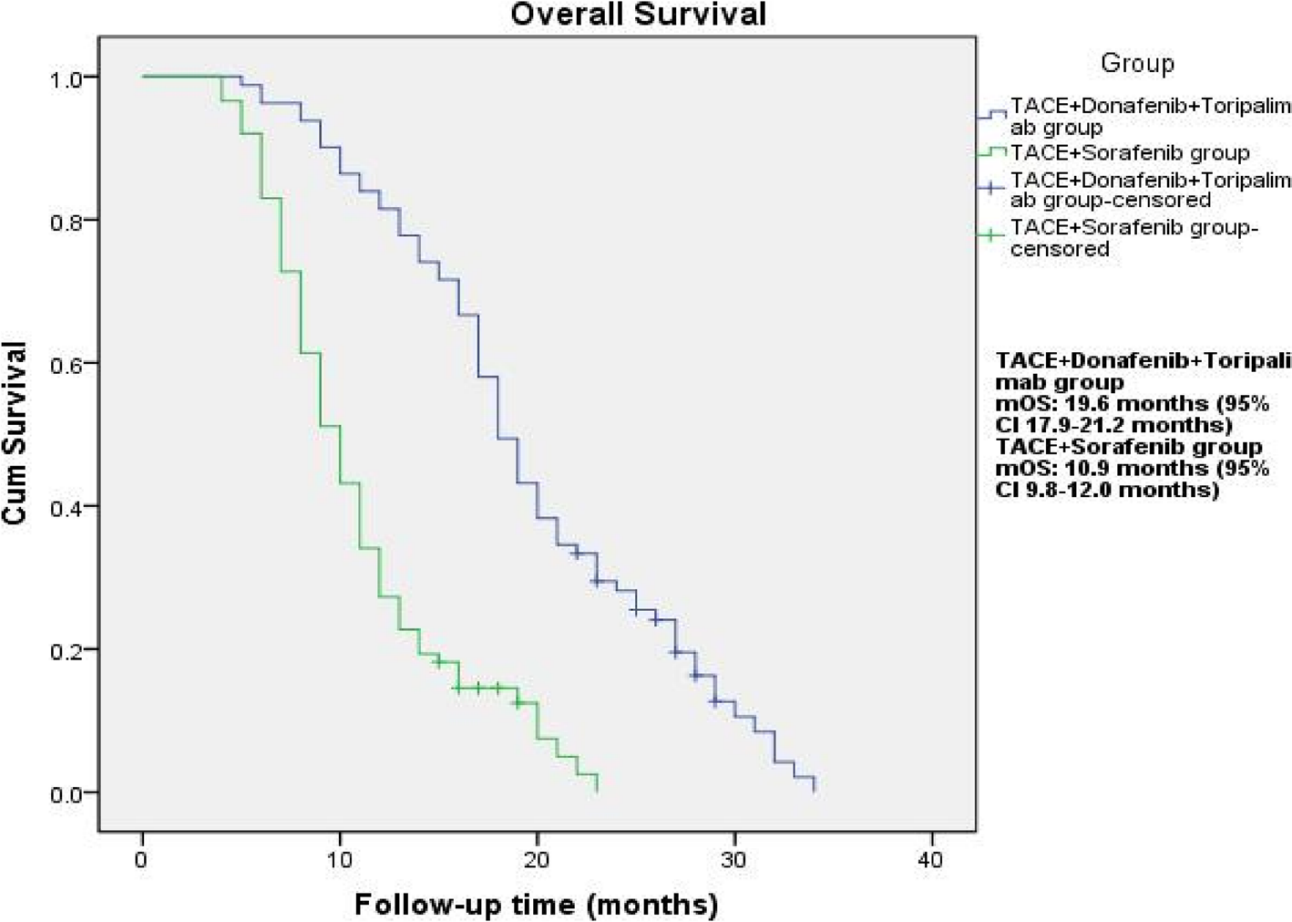
Overall survival of patients in two groups. mOS: TACE+Donafenib+Toripalimab group, 19.6 months (95% CI: 17.9-21.2 months); TACE+Sorafenib group, 10.9 months (95% CI 9.8-12.0 months)

The median PFS was longer in the TACE + Donafenib + Toripalimab group compared to the TACE + Sorafenib group (10.9 months vs. 7.0 months), and the difference was statistically significant (*P* < 0.001, Fig. 1). The median OS was longer in the TACE + Donafenib + Toripalimab group compared to the TACE + Sorafenib group (19.6 months vs. 10.9 months), and the difference was statistically significant (*P* < 0.001, Fig. 2).

### Incidence of treatment-related adverse events in the two groups (see Table 5)

**Table 5:** Incidence of Adverse Events in the Two Patient Groups after Treatment

	Group				Chi-Square Tests (*p*-value)	
Adverse events	TACE + Donafenib + Toripalimab group (*N* = 81)		TACE + Sorafenib group (*N* = 88)			
	All grades, n (%)	Grade 3/4, n (%)	All grades, n (%)	Grade 3/4, n (%)	All grades	Grade 3/4
Abdominal pain	61(75.3%)	24(29.6%)	70(79.5%)	35(39.8%)	0.582	0.197
Fever	50(61.7%)	20(24.7%)	59(67.0%)	24(27.3%)	0.521	0.729
Vomiting	46(56.8%)	20(24,7%)	49(55.7%)	19(21.6%)	0.885	0.716
Hypertension	23(28.4%)	4(4.9%)	28(31.8%)	13(14.8%)	0.738	0.041
Diarrhea	6(7.4%)	0(0.0%)	16(18.2%)	3(3.4%)	0.042	0.247
Hand-foot syndrome	10(12.3%)	1(1.2%)	23(26.1%)	4(4.5%)	0.032	0.370
Skin rash	11(13.6%)	0(0.0%)	14(15.9%)	2(2.3%)	0.829	0.498
Fatigue	10(12.3%)	1(1.2%)	15(17.0%)	1(1.1%)	0.516	0.953
Anorexia	8(9.9%)	1(1.2%)	12(13.6%)	0(0.0%)	0.484	0.479
Gastrointestinal Hemorrhage	2(2.5%)	0(0.0%)	4(4.5%)	1(1.1%)	0.683	0.336

Compared to the TACE + Donafenib + Toripalimab group, the TACE + Sorafenib group had a higher incidence of grade 3–4 hypertension (14.8% vs. 4.9%, *P* = 0.041), a higher incidence of diarrhea (all grades) (18.2% vs. 7.4%, *P* = 0.042), and a higher incidence of hand-foot syndrome (all grades) (26.1% vs. 12.3%, *P* = 0.032). There were no statistically significant differences (*P* > 0.05) between the two groups in terms of abdominal pain, fever, vomiting, rash, fatigue, anorexia, and gastrointestinal hemorrhage.

### Discussion

Hepatocellular carcinoma (HCC) is one of the most common primary malignant tumors of the liver, and its incidence and mortality rates are increasing. Due to the highly malignant and complex nature of HCC, its treatment has been a focus of clinical research. Transarterial chemoembolization (TACE) has become a commonly used method for treating unresectable HCC. On one hand, the chemotherapeutic drugs injected through the catheter can induce tumor cell apoptosis and inhibit tumor cell proliferation. At the same time, embolizing the tumor-feeding arteries can lead to ischemia, hypoxia, and necrosis of the tumor tissue [28]. The theoretical basis of TACE treatment is that the normal liver tissue has a dual blood supply, with the portal vein as the main supply. Therefore, performing arterial chemoembolization of liver tumors has minimal impact on normal liver tissue [29]. Jinpeng Li et al. [30] reported on 172 HCC patients who underwent TACE treatment, and the results showed that the objective response rate (ORR) at 2, 4, and 6 months after treatment was 78.7%, 71.6%, and 63.2%, respectively, and the disease control rate (DCR) was 95.3%, 92.1%, and 85.9%, respectively. However, TACE embolization can cause tumor tissue ischemia and hypoxia, leading to upregulation of hypoxia-inducible factor-1 alpha (HIF-1α) and subsequent upregulation of levels of VEGF, FGF, and other factors, which may contribute to tumor recurrence and metastasis [31, 32]. Adriana Sergio et al. [33] reported that when TACE is not completely effective, it may induce a significant neovascularization response, such as increased levels of VEGF and b-FGF after treatment, which can impact patient survival.

Donafenib can simultaneously inhibit the activity of various receptor tyrosine kinases, such as VEGFR and PDGFR, and directly inhibit various Raf kinases, as well as downstream Raf/MEK/ERK signaling pathways. It inhibits tumor cell proliferation and tumor angiogenesis, exerting a dual inhibitory and multi-target blocking anti-tumor effect [34]. Therefore, the combination of TACE and donafenib has a synergistic effect in terms of mechanisms. Shukui Qin et al. [35] reported on 668 HCC patients who were randomly assigned to the donafenib group (328 cases) and the sorafenib treatment group (331 cases), and the median overall survival (mOS) in the donafenib treatment group was significantly longer than that in the sorafenib treatment group (FAS; 12.1 vs. 10.3 months; hazard ratio, 0.831; *P* = 0.0245).

TACE combined with immune checkpoint inhibitors is a combination strategy used for comprehensive treatment of hepatocellular carcinoma (HCC), and its mechanisms and principles are as follows [36]: TACE blocks the blood supply from the hepatic artery, which not only directly kills tumor cells but also releases tumor-associated antigens (TAAs) and inflammatory mediators, promoting activation of the immune system. Ahmed Montasser et al. [37] reported on 82 HCC cases that underwent surgical treatment, with 32 cases receiving prior TACE and 50 cases not receiving TACE. The study results showed increased expression of PD-1 and PD-L1 in HCC after TACE treatment. The mechanism of immune checkpoint inhibitors [38, 39]: Immune checkpoint inhibitors such as PD-1 inhibitors and CTLA-4 inhibitors can block the signaling of immune checkpoint receptors on the surface of liver cancer cells and their ligands, restoring the activation and attacking capability of immune cells. These inhibitors can relieve immune suppression and enhance the recognition and killing of liver cancer cells by T cells and natural killer (NK) cells. By combining TACE and immune checkpoint inhibitors, a synergistic effect of local treatment and systemic immune activation can be achieved. The release of tumor antigens by TACE can stimulate immune cell responses, while immune checkpoint inhibitors can eliminate immune suppression and enhance the activity of immune cells. This combination treatment strategy helps promote antigen-specific immune responses against hepatocellular carcinoma and improve treatment outcomes. Brett Marinelli et al. [40] reported that patients receiving TACE combined with immune therapy had longer median progression-free survival (mPFS) and median overall survival (mOS) compared to those receiving single-agent immune therapy, with values of 8.8 months vs. 3.7 months (*p* < 0.01) and 35.1 months vs. 16.6 months (*p* = 0.12), respectively. Caihua Zhu et al. [41] reported on 20 patients with advanced-stage liver cancer who received neoadjuvant TACE combined with PD-1 inhibitor bridging to surgical treatment. The objective response rate (ORR) of neoadjuvant treatment was 75.0%, and the disease control rate (DCR) was 100.0%. Fourteen patients (70.0%) achieved successful downstaging (converted to CNLC Stage I).

Toripalimab is a fully human monoclonal antibody against the PD-1 receptor [42], which can block the binding of PD-1 on T lymphocytes to PD-L1 on tumor cells, relieving immune suppression of tumor cells and allowing immune cells to exert anti-tumor immune effects and kill tumor cells [43]. ZhiCheng Lai et al. [44] reported on 36 patients with advanced HCC who received lenvatinib, toripalimab, and FOLFOX-HAIC, with a median progression-free survival (mPFS) of 10.4 months and median overall survival (mOS) of 17.9 months. Wei-Feng Qu et al. [45] reported on 51 unresectable HCC patients, with 30 patients receiving triple combination therapy (t-CT: lenvatinib, TACE, plus toripalimab) and 20 patients receiving dual combination therapy (d-CT: lenvatinib plus TACE). Compared to d-CT, t-CT had higher objective response rate (ORR) (76.7% vs. 47.6%, *P* = 0.042) and disease control rate (DCR) (90.0% vs. 57.1%, *P* = 0.042).

Currently, many scholars have utilized transcatheter arterial chemoembolization (TACE) combined with molecular targeted therapy and immune checkpoint inhibitors for the treatment of unresectable hepatocellular carcinoma (uHCC), achieving significant therapeutic efficacy. Shuping Qu et al. [46] reported a study involving 110 uHCC patients, among whom 56 patients received combination therapy (TACE Combined With Lenvatinib Plus PD-1 Inhibitors) and 54 patients received TACE alone. Compared to the TACE group, the combination therapy group demonstrated a higher objective response rate (67.9% vs. 29.6%, *p* < 0.001), longer median progression-free survival (11.9 months vs. 6.9 months, *p* = 0.003), and longer median overall survival (23.9 months vs. 15.3 months, *p* < 0.001). Mingyue Cai et al. [47] reported a study involving 81 patients with advanced HCC, among whom 41 received TACE combined with lenvatinib plus PD-1 inhibitor and 40 received TACE combined with lenvatinib. Compared to the TACE combined with lenvatinib group, the TACE combined with lenvatinib plus PD-1 inhibitor group exhibited extended median overall survival (16.9 months vs. 12.1 months, *p* = 0.009), prolonged median progression-free survival (7.3 months vs. 4.0 months, *p* = 0.002), higher objective response rate (56.1% vs. 32.5%, *p* = 0.033), and higher disease control rate (85.4% vs. 62.5%, *p* = 0.019). Yan-Jun Xiang et al. [48] reported that in patients with BCLC stage B HCC, TACE combined with PD-1 inhibitors and lenvatinib treatment significantly improved clinical outcomes compared to TACE combined with PD-1 inhibitors alone, while maintaining controllable safety.

However, currently, there are multiple options for molecular targeted drugs and immune checkpoint inhibitors used in the treatment of hepatocellular carcinoma, and whether different drug combination regimens can achieve favorable therapeutic effects, as well as which combination regimen can provide greater survival benefits for HCC patients, are directions that many scholars have been exploring. This study found that the TACE + Donafenib + Toripalimab group had longer median progression-free survival and overall survival than the TACE + Sorafenib group, with statistical differences (*p* < 0.001).

In addition, we observed that the TACE combined with Donafenib and Toripalimab group showed advantages in terms of objective response rate (ORR) and disease control rate (DCR) (66.7% vs. 38.6%, 82.6% vs. 68.2%, *p* < 0.05). This suggests that this combination therapy regimen can better control tumor growth and progression, thereby improving treatment response and disease control level in patients. The results of this study indicate that patients in the TACE combined with Sorafenib group had relatively less improvement in terms of survival. Sorafenib, as a targeted therapy drug, has been widely used in the treatment of HCC. However, its efficacy as a single agent is not satisfactory, which is consistent with the results of some clinical studies. Shou Wu Lee et al. [49] reported a study involving 53 BCLC stage C HCC patients who received TACE combined with Sorafenib treatment, with a median time to progression (mTTP) of 6.42 months and median overall survival (mOS) of 11.21 months. Yashwant Patidar et al. [50] reported a study involving 31 advanced HCC patients treated with TACE combined with Sorafenib, with a disease control rate (DCR) of 44.9%, mTTP of 4.6 months, and mOS of 10.1 months. Therefore, our results further support the effectiveness of TACE combined with Donafenib and Toripalimab as a treatment strategy for unresectable HCC.

Regarding safety assessment, we observed that patients in the TACE combined with Donafenib and Toripalimab group had lower incidence rates of high-grade hypertension, diarrhea (all grades), and hand-foot syndrome (all grades) compared to the TACE combined with Sorafenib group. The incidence rates and severity of other common adverse events were comparable between the two groups. This indicates that while providing patients with more treatment options, the combination therapy regimen did not significantly increase adverse effects. The reason for this may be that donafenib is a deuterated derivative of sorafenib, which enhances its stability and reduces susceptibility to hepatic drug-metabolizing enzymes, leading to increased plasma exposure and decreased formation of toxic metabolites [51, 52]. The improved pharmacokinetic profile of donafenib may explain its improved safety characteristics compared to sorafenib, resulting in a lower incidence of adverse reactions. Shukui Qin et al. [35] reported that patients receiving donafenib treatment had a significantly lower incidence of grade 3 or higher drug-related adverse events compared to sorafenib (38% vs. 50%; *p* = 0.0018). Jingrui Liu et al. [53] reported a study involving 27 eligible advanced HCC patients treated with oral donafenib. They were randomly divided into 200 mg and 300 mg bid groups, and both groups showed good safety and tolerability of donafenib, with most adverse events being grade 1 or grade 2.

It is important to note that this study has several limitations. Firstly, due to its retrospective design, there may be selection bias and limitations in data acquisition. Secondly, this study is single-center, which may limit the generalizability and external validity of the results. Future studies could employ a multicenter, prospective design to further validate our findings.

## Conclusion

The TACE combined with Donafenib and Toripalimab group showed higher ORR and DCR, as well as longer PFS and OS compared to the TACE combined with Sorafenib group. The TACE combined with Donafenib and Toripalimab group did not increase the incidence of treatment-related adverse events. In conclusion, the results of this study suggest that the combination of TACE with Donafenib and Toripalimab demonstrates good efficacy and safety in the treatment of unresectable HCC patients. This combination therapy regimen may be a feasible option to improve the prognosis of unresectable HCC patients. However, further research is still needed to comprehensively evaluate its efficacy and safety and optimize treatment strategies for personalized HCC therapy.

## Data Availability

The datasets used during the current study are available from the corresponding author upon request.
